# Supporting Cognition With Modern Technology: Distributed Cognition Today and in an AI-Enhanced Future

**DOI:** 10.3389/frai.2022.908261

**Published:** 2022-07-14

**Authors:** Sandra Grinschgl, Aljoscha C. Neubauer

**Affiliations:** Institute of Psychology, University of Graz, Graz, Austria

**Keywords:** technology, artificial intelligence (AI), distributed cognition, cognitive offloading, trust

## Abstract

In the present article, we explore prospects for using artificial intelligence (AI) to distribute cognition *via* cognitive offloading (i.e., to delegate thinking tasks to AI-technologies). Modern technologies for cognitive support are rapidly developing and increasingly popular. Today, many individuals heavily rely on their smartphones or other technical gadgets to support their daily life but also their learning and work. For instance, smartphones are used to track and analyze changes in the environment, and to store and continually update relevant information. Thus, individuals can offload (i.e., externalize) information to their smartphones and refresh their knowledge by accessing it. This implies that using modern technologies such as AI empowers users *via* offloading and enables them to function as always-updated knowledge professionals, so that they can deploy their insights strategically instead of relying on outdated and memorized facts. This AI-supported offloading of cognitive processes also saves individuals' internal cognitive resources by distributing the task demands into their environment. In this article, we provide (1) an overview of empirical findings on cognitive offloading and (2) an outlook on how individuals' offloading behavior might change in an AI-enhanced future. More specifically, we first discuss determinants of offloading such as the design of technical tools and links to metacognition. Furthermore, we discuss benefits and risks of cognitive offloading. While offloading improves immediate task performance, it might also be a threat for users' cognitive abilities. Following this, we provide a perspective on whether individuals will make heavier use of AI-technologies for offloading in the future and how this might affect their cognition. On one hand, individuals might heavily rely on easily accessible AI-technologies which in return might diminish their internal cognition/learning. On the other hand, individuals might aim at enhancing their cognition so that they can keep up with AI-technologies and will not be replaced by them. Finally, we present own data and findings from the literature on the assumption that individuals' personality is a predictor of trust in AI. Trust in modern AI-technologies might be a strong determinant for wider appropriation and dependence on these technologies to distribute cognition and should thus be considered in an AI-enhanced future.

## Introduction

Today, modern technologies are an indispensable part of peoples' lives and it is hard to image living without smartphones and other technical gadgets. Currently, around 1.3 billion smartphones are sold worldwide per year (idc.com, 2022) and, for instance, in Austria about 83% of individuals above 15 years own a smartphone (KMU Forschung Austria, 2020). People are using their technical devices for several reasons, such as staying in contact, using the internet, taking pictures, or playing games. Furthermore, technical devices can be used to externalize cognitive processes, which is referred to as *cognitive offloading* (Risko and Gilbert, 2016). Individuals offload cognitive processes by, for instance, relying on navigation applications instead relying on one's own spatial abilities or by storing appointments or shopping lists in their smartphones instead of memorizing them. Also, modern technologies can be used to access up-to-date knowledge and thus individuals do not need to rely on outdated, internally memorized information. Indeed, the majority of adults indicates using technical devices regularly or even very often as external memory stores (Finley et al., 2018). Also, empirical studies show that individuals flexibly distribute cognitive demands between internal and external resources when solving problems (e.g., Cary and Carlson, 2001). Distributed cognition using modern technologies should thereby facilitate task performance.

Besides the mentioned basic applications of a technical device, also smart applications that are based on artificial intelligence (AI) might be used for offloading. For instance, smart speakers could be used to store appointments and to be reminded of them. Due to the effortless interaction with such smart applications distributed cognition might reach a new all-time high in the coming years. Here, we aim at discussing this development of distributed cognition with modern technologies. First, we give an overview of recent findings regarding cognitive offloading. We discuss determinants that foster the offloading of cognitive processes in modern technologies as well as possible benefits and risks of offloading. Second, we provide an outlook on how distributed cognition (and thus cognitive offloading) might change in the future–when AI-technologies are used on a daily basis by many people–especially with regard to the educational sector. Furthermore, we discuss whether distributed cognition will actually increase in the future or whether people will rather aim at enhancing their internal cognitive abilities. One crucial factor for using AI-technologies to distribute cognition might be peoples' trust in these technologies. Therefore, we also describe personality traits as potential predictors of trust in AI by summarizing findings from the literature and presenting our own data. Finally, we argue for more psychological research that could support and inform the development of modern technologies.

## Overview of Cognitive Offloading with Modern Technologies

While cognitive offloading comes in many different forms (e.g., for short term memory offloading see Meyerhoff et al., 2021; for navigation offloading see Fenech et al., 2010), investigations on the offloading of cognitive processes into modern technology can be summarized into two lines of research: (1) the determinants of cognitive offloading and (2) the consequences of offloading behavior.

Modern technologies could be used for offloading whenever they are available, but offloading might be applied more or less depending on certain conditions. On the one hand, offloading was shown to depend on external factors such as the design of technical tools (e.g., Gray et al., 2006; Grinschgl et al., 2020) or characteristics of the to-be-processed information (e.g., Schönpflug, 1986; Hu et al., 2019). Regarding tool design, studies observed that individuals offload more cognitive processes when the offloading process (i.e. the interaction with a technical tool) is fast vs. associated with temporal delays (e.g., Gray et al., 2006; Waldron et al., 2011; Grinschgl et al., 2020). Similarly, also when interacting with technical tools requires less vs. more operational steps offloading increases (e.g., O'Hara and Payne, 1998; Cary and Carlson, 2001). Furthermore, Grinschgl et al. (2020) observed that when participants performed a task on a tablet using its touch function, they offloaded more working memory processes than when using a computer mouse. Regarding the characteristics of the to-be-processed information, it was shown that offloading increases when a task and accompanying information is more complex, relevant, or difficult (e.g., Schönpflug, 1986; Hu et al., 2019). Additionally, a larger amount of to-be-processed information fosters offloading behavior (e.g., Gilbert, 2015a; Arreola et al., 2019). Overall, these external factors suggest that the distribution of cognitive processes on internal and external resources depends on situational cost-benefit considerations (e.g., Gray et al., 2006; Grinschgl et al., 2020).

On the other hand, internal factors such as individuals' cognitive abilities and metacognitive beliefs can impact offloading. Studies observed more offloading when one's working memory capacity is lower (e.g., Gilbert, 2015b; Meyerhoff et al., 2021). Moreover, individuals commonly offload more when they believe that their internal performance is worse (Gilbert, 2015b; Boldt and Gilbert, 2019; but see Grinschgl et al., 2021a for conflicting results). To our best knowledge, an investigation of other individual differences with regard to cognitive offloading is still lacking (e.g., there is no research on the interplay between personality and offloading).

Together, these determinants of offloading behavior suggest that individuals do not maximally offload under all circumstances, but instead especially the easy and fast access to modern technology fosters offloading (e.g., Grinschgl et al., 2020). With an increase in offloading due to modern technologies, the question arises whether offloading is accompanied by positive and/or negative consequences. Cognitive offloading was shown to improve immediate task performance by accelerating it and/or reducing errors (e.g., Boldt and Gilbert, 2019; Grinschgl et al., 2021b). Furthermore, studies showed that offloading improves simultaneous secondary task performance (Grinschgl et al., 2022) and later performance of unrelated tasks (Storm and Stone, 2015; Runge et al., 2019). Hence, it is assumed that cognitive offloading releases internal cognitive resources that can be devoted to other, simultaneous or subsequent tasks. Furthermore, the retrieval and storage of information using modern technologies might help individuals to refresh their internal knowledge and thus to act as always-updated knowledge professionals.

Importantly, cognitive offloading is also accompanied by risks. In three experiments, Grinschgl et al. (2021b) observed a trade-off for cognitive offloading: while the offloading of working memory processes increased immediate task performance, it also decreased subsequent memory performance for the offloaded information. Similarly, the offloading of spatial processes by using a navigation device impairs spatial memory (i.e., route learning and subsequent scene recognition; Fenech et al., 2010). Thus, information stored in a technical device might be quickly forgotten (for an intentional/directed forgetting account see Sparrow et al., 2011; Eskritt and Ma, 2014) or might not be processed deeply enough so that no long-term memory representations are formed (cf. depth of processing theories; Craik and Lockhart, 1972; Craik, 2002). In addition to detrimental effects of offloading on (long-term) memory, offloading hinders skill acquisition (Van Nimwegen and Van Oostendorp, 2009; Moritz et al., 2020) and harms metacognition (e.g., Fisher et al., 2015, 2021; Dunn et al., 2021); e.g. the use of technical aids can inflate one's knowledge. In Dunn et al. (2021), the participants had to answer general knowledge questions by either relying on their internal resources or additionally using the internet. Metacognitive judgments showed that participants were overconfident when they are allowed to use the internet. Similarly, Fisher et al. (2021) concluded that searching for information online leads to a misattribution of external information to internal memory.

To summarize, cognitive offloading is accompanied by both–benefits and risks. While it can improve immediate task performance, it might also be accompanied by detrimental long-term effects. However, the investigated time-frames were rather short (effects over hours or days). Thus, it remains unclear how offloading might impact cognition over the lifespan (for a discussion see Cecutti et al., 2021). While these authors see humans' development with modern technologies rather positive, others pose modern technologies as a threat for humans (e.g., Carr, 2008; Spitzer, 2012). If distributing cognition actually is a blessing or a threat for human cognition cannot be answered here, but we will provide a brief outlook on how distributed cognition might be affected by the rise of AI.

## Outlook on Distributed Cognition in an AI-Enhanced Future

As the use of many AI-technologies such as smart speakers appears quite effortless, they might be used to easily store appointments, take notes, retrieve up-to-date knowledge, or to perform other cognitive tasks (e.g., calculating, navigating). AI-technologies might thus be the “future” of distributed cognition by replacing classical offloading tools. With this prospect, it is important to consider how the omnipresence of AI as offloading tools might impact human cognition and how we–as humans–might foster a worthwhile integration of AI into our life.

To date, cognitive offloading research has shown positive and negative consequences of using modern technology to distribute demands on internal and external resources. However, these studies did not target the use of AI-technologies as offloading tools, but standard technologies such as tablets/computers without AI applications. To our best knowledge, research investigating distributed cognition in the context of AI is lacking. While we see a high potential for new studies on this matter, we also think that previous results can be transferred to the current and future use of AI. Therefore, AI-technologies should be used with caution as they might diminish cognitive abilities such as learning and memory–consequences that are especially relevant when it comes to children's education.

Studies suggest that even young children offload cognitive processes instead of completely relying on their internal resources (e.g., Armitage et al., 2020; Bulley et al., 2020). Thus, already in crucial learning phases during childhood tools are used to distribute cognitive demands onto internal and external resources. Such offloading behavior might increase with the ever earlier access to modern AI-technologies in smartphones and computers. Especially regarding education, it must be discussed whether there should be a “ban” of cognitive offloading due to potential detrimental effects thereof or whether students need to learn how to properly use technical tools without causing harm for their cognition (cf. Bearman and Luckin, 2020; Dawson, 2020). In line with these authors, we advocate to teach students how to use technical devices so that they satisfy their needs but to not (unintentionally) harm cognition. For instance, students need to learn how to differentiate between their own knowledge and externally stored knowledge, so that the effect of inflated knowledge is avoided. Furthermore, students should be made aware of their offloading behavior and that they won't be able to access their technical tools in critical situations such as during exams. This is especially important as a study showed that cognitive offloading was not detrimental for long-term memory when participants were forced to offload but also were instructed to internally memorize the relevant information (Grinschgl et al., 2021b). Thus, offloading is not always detrimental for building long-term memory representations and instead detriments of offloading might be compensated by proper learning instructions. Additionally, modern (AI-)technologies can benefit education by providing students with automated feedback to improve learning (Bearman and Luckin, 2020). Hence, the availability of modern (AI)-technologies is accompanied by both benefits and risks.

One alternative to strongly relying on AI to perform demanding tasks, might be the enhancement of one's own cognition. Especially the Transhumanism movement in philosophy proposes the enhancement of human cognition, such as intelligence, so that we are able to solve global problems (e.g., the climate crisis; Liao et al., 2012; Sorgner, 2020). This enhancement should be achieved by enhancement methods such as taking smart drugs, stimulating the brain, or modifying genes (Bostrom and Sandberg, 2009). However, so far the effects of most enhancement methods are at best moderate (Hills and Hertwig, 2011; Jaušovec and Pahor, 2017). The future might bring new possibilities to foster human enhancement and this might enable humans to compete with AI (for a discussion on the implications of human enhancement and AI see Neubauer, 2021).

As human enhancement is not a promising strategy to become smarter yet, individuals might rather rely on the available technologies to improve their performance. As outlined before, individuals do not rely on technical tools all the time, but the distribution of cognitive processes onto internal and external resources rather depends on factors such as tool design, one's abilities, or metacognitive beliefs. Another factor that might strongly influence the reliance on AI might be the trust in these technologies. In a recent review, Matthews et al. (2021) identified trust as a major factor when it comes to human-machine interaction and suggested that trust in AI should be systematically investigated as humans are approaching a technology-enhanced future. Trust can be seen as the specific beliefs about technology and the willingness to rely on technology in risky situations (Siau and Wang, 2018). Thus, trust might determine if and how individuals interact with AI (see also Glikson and Woolley, 2020; Chong et al., 2022) for distributed cognition. The question arises whether there are individual differences when it comes to trusting AI. An important source for individual differences might be individuals' personality such as the Big 5 traits (Hoff and Bashir, 2015; Matthews et al., 2021). Therefore, we briefly summarize research investigating personality traits as potential predictors of trust in AI and present our own data on this matter.

On one hand, several studies investigated trust regarding specific AI-technologies. Li et al. (2020) consistently observed correlations between openness and trust in automated driving. While the participants showed lower trust in automated driving with higher openness in a questionnaire, they also showed a higher monitoring frequency, more frequent, and earlier and longer “take overs” in an automated driving simulator. Individuals high in openness might strive for more intellectually demanding tasks and thus do not heavily rely on automated systems (but see Zhang et al., 2020, for conflicting results). Additionally, higher extraversion was related to less trust in the questionnaire but no other effects were observed (Li et al., 2020). In contrast, Kraus et al. (2021) did not observe a relationship between openness and trust in automated driving, but indirect effects of neuroticism, extraversion, and agreeableness. In a path model, higher neuroticism was related to less affinity for technology. A higher affinity for technology was positively related to trust in automated driving (see also Zhang et al., 2020). Moreover, higher extraversion and agreeableness were related to more interpersonal trust which was related to more trust in automated driving. These findings suggest that there is a common factor underlying both trust in humans and trust in automated systems (Kraus et al., 2021). Besides automated driving, Sharan and Romano (2020) investigated trust in AI by providing participants with suggestions from an AI-algorithm when making decisions in a card game and found none of the Big 5 traits correlated to any trust indicators.

Other studies assessed general trust in AI- (or related) technologies. For instance, Chien et al. (2016) observed that higher agreeableness and conscientiousness were related to more trust in automated systems, with no significant relationships for the other Big 5 traits. Merrit and Ilgen (2008) observed that extraversion is positively related to the propensity to trust machines; additionally, age was negatively related to trust in machines. In our own, exploratory study (*N* = 467; for details see Supplementary Material), we assessed general trust in AI by a 7-item questionnaire. Additionally, we measured participants sociodemographic data (age, gender, and education), Big 5 traits and facets, and their interpersonal trust. The correlations of these variables with general trust in AI can be found in Table 1. In contrast to single previous studies (e.g., Chien et al., 2016), we did not observe any relationships between trust in AI and Big 5 traits as well as their facets. Additionally, interpersonal trust was not related to trust in AI. We, thus, cannot confirm the findings of Kraus et al. (2021), suggesting trust in others and in technology are positively correlated. However, in line with Merrit and Ilgen (2008), we observed a negative relationship between age and trust in AI as well as no gender effects. Older adults seem to have less trust in AI, potentially due to less experience with these systems and modern technology in general (but see Zhang et al., 2020, for diverging results).

**Table 1:** Correlations between sociodemographic data, Big 5 traits and facets, interpersonal trust, and general trust in AI.

	**Trust in AI**
**Age**	−0.17^*^
**Gender**	0.04
**Education**	0.09
**Openness**	−0.02
Openness–aesthetics	−0.01
Openness–ideas	−0.02
**Agreeableness**	0.02
Agreeableness–altruism	0.03
Agreeableness–concession	0.03
**Conscientiousness**	−0.12
Conscientiousness order	−0.12
Conscientiousness–self-discipline	−0.11
**Extraversion**	0.06
Extraversion–assertiveness	0.06
Extraversion–activity	0.04
**Neuroticism**	−0.07
Neuroticism–anxiety	−0.05
Neuroticism–depression	−0.08
**Interpersonal trust**	0.08

*N = 467; for gender N = 466 because of the exclusion of one non-binary participant; women are coded as 0, men are coded as 1; significance levels are Bonferroni-Holm corrected;*

* *p < 0.05*.

To summarize, results regarding personality traits as predictors of trust in AI seem rather inconsistent and depend on the targeted AI-technology (see also Schäfer et al., 2016). We thus urge for more systematic research to identify personality traits and other factors that might predict the trust in and use of AI-technologies in general but also for distributed cognition more specifically. Besides personality, other factors might play a major role for trusting and using AI. For instance, the perceived usability of AI-technologies, computer anxiety, task characteristics, transparency, or perceived intelligence of AI might determine if and how AI-technologies are used (for an overview and further differentiation see Glikson and Woolley, 2020; Kaplan et al., 2021; Matthews et al., 2021).

## Discussion

While smartphones and other technical gadgets are already frequently used to distribute cognition, the rise of easy-to-use AI-technologies might further foster the offloading of cognitive processes. This development urges a need to investigate consequences of using AI for distributed cognition and to inform the public about these consequences. Although AI is already often discussed by information scientists, politics, and the general public, psychologists and psychological findings are barely integrated into these discussions. We see a high potential for psychological research to inform the public about potentials of modern technologies but also about accompanied risks. Moreover, identifying individuals that would easily rely on AI (e.g., due to a high trust in these systems) could help in specifically targeting these individuals when it comes to potential negative consequences of heavily relying on technology. Thus, we argue for more individual differences research to systematically investigate which factors (e.g., personality) predict trust in and use of AI. Growing up with modern technologies will likely affect our cognition as well as our attitude toward technologies. Such findings should be considered both when designing modern technologies and when using them in different situations (e.g., in private life, educational settings)–already now and in an AI-enhanced future.

## Data Availability Statement

The raw data supporting the conclusions of this article will be made available by the authors upon request, without undue reservation.

## Ethics Statement

The studies involving human participants were reviewed and approved by Ethics Committee of the University Graz. The patients/participants provided their written informed consent to participate in this study.

## Author Contributions

SG: conceptualization, methodology, data curation, data analysis, and writing—original draft. AN: conceptualization, methodology, and writing—review and editing. All authors contributed to the article and approved the submitted version.

## Funding

The authors acknowledge the financial support by the University of Graz for the Open Access Publication.

## Conflict of Interest

The authors declare that the research was conducted in the absence of any commercial or financial relationships that could be construed as a potential conflict of interest.

## Publisher's Note

All claims expressed in this article are solely those of the authors and do not necessarily represent those of their affiliated organizations, or those of the publisher, the editors and the reviewers. Any product that may be evaluated in this article, or claim that may be made by its manufacturer, is not guaranteed or endorsed by the publisher.
