# Supplementary material for: Supporting Cognition With Modern Technology: Distributed Cognition Today and in an AI-Enhanced Future
Source: Front Artif Intell. 2022 Jul 14;5:908261. doi: 10.3389/frai.2022.908261 (PMC9329671; doi:10.3389/frai.2022.908261)
Supplement: Supplementary file 1 [file Data_Sheet_1.docx]

Supplementary Material

# Supplementary Material - Short description of study:

In April and May 2021, participants could participate in this study by filling out several online questionnaires. Besides questionnaires on trust in AI, sociodemographic data, the Big 5 traits and interpersonal trust, the study also included several other questionnaires (see procedure) as multiple, unrelated research questions were investigated with this study.

Participants:

In total, 467 participants completed the online study on their own technical device (e.g., their laptop). The participants were between 18 and 77 years old (*M* = 31.32, *SD* = 14.89; 290 women, 179 men, 1 non-binary) and needed to speak German fluently. Regarding the educational level of our sample, most of the participants had either obtained their A-levels (i.e. “Matura/Abitur”; 259 participants) or a university degree (113 participants). 65 participants had completed an apprenticeship or a vocational school, 18 participants a secondary school, 11 participants a lower secondary school and one participant did not obtain a school diploma. All participants gave written informed consent prior to participating. The study was following the ethical principles of the Declaration of Helsinki.

Procedure:

In the following procedure, all questionnaires that were included in this online study are listed:

- Informed consent and information on data protection
- **Soziodemographic data (age, gender, education)**
- **Big 5 personality questionnaire**
- **Interpersonal trust questionnaire**
- Interpersonal reactivity index
- Thrill & adventure seeking scale
- Engagement with AI
- **Trust in AI**
- Likability of AI
- Knowledge about AI
- Self-driving car acceptance
- Use of social media
- Use of instagram
- Narcissism questionnaire
- Hostility questionnaire
- Covid-19 vaccination willingness

Bold questionnaires are relevant for the present research question and are described in more detail below. The completion of the whole study took about 20 minutes. All questionnaires were administered in German.

Questionnaires:

We briefly describe the questionnaires that were used for the exploratory analyses of the relationships between sociodemographic data, Big 5 traits, interpersonal trust, and trust in AI. We will not further describe the other questionnaires that were included in the study to investigate different research questions.

Big 5 personality questionnaire. To assess the Big 5 traits and two sub-facets for each trait, we used the Big Five Inventory by Rammstedt and Danner (2016). This questionnaire includes 45 items that needed to be answered on a 5-point Likert scale from “1 = strongly disagree” to “5 = strongly agree”. Each Big 5 trait is measured by eight to ten items and trait scores were averaged across items. The internal consistencies of all traits were acceptable or good (Cronbach’s α >= .78). In addition, for each Big 5 trait we derived two sub-facets. For these facets, internal consistencies were a bit lower (Cronbach’s α >= .54), which is not surprising as they comprise less items (see Rammstedt & Danner, 2016).

Interpersonal trust questionnaire. We measured participants’ interpersonal trust by using the short scale of interpersonal trust (KUSIV3, Beierlein et al., 2012). In this questionnaire, the participants answer three questions on a 5-point Likert scale from “1 = don’t agree at all” to “5 = agree completely”. The answers to these three questions are averaged to derive a score for interpersonal trust. The internal consistency of this questionnaire was acceptable (Cronbach’s α = .78).

Trust in AI. To measure participants’ general trust in AI-technologies, we adapted and translated questionnaires from previous studies (Merrit, 2011; Merrit et al., 2013). The adapted questionnaire included seven questions, such as “I have confidence in the advice given by an AI”. The participants answered these questions on a 5-point Likert scale ranging from “1 = strongly disagree” to “5 = strongly agree”. We averaged the answers across the seven questions as a score of general trust in AI. The internal consistency was good (Cronbach’s α = .86).

References:

Beierlein, C., Kemper, C. J., Kovaleva, A., & Rammstedt, B. (2012). Kurzskala zur Messung

des zwischenmenschlichen Vertrauens: die Kurzskala Interpersonales Vertrauen

(KUSIV3). *GESIS – Leibniz-Institut für Sozialwissenschaften*.

<https://nbn-resolving.org/urn:nbn:de:0168-ssoar-312126>

Merritt, S. M. (2011). Affective Processes in Human-Automation Interactions. *Human Factors*, *53,* 356 – 370. <https://doi.org/10.1177%2F0018720811411912>

Merritt, S. M., Heimbaugh, H., LaChapell, J., & Lee, D. (2013). I Trust It, but I Don’t Know

Why: Effect of Implicit Attitudes Toward Automation on Trust in an Automated System. *Human Factors*, *55,* 520-534. <https://doi.org/10.1177%2F0018720812465081>

Rammstedt, B., & Danner, D. (2016). Die Facettenstruktur des Big Five Inventory (BFI):

Validierung für die Deutsche Adaptation des BFI*. Diagnostica, 63*, 70-84.

<https://doi.org/10.1026/0012-1924/a000161>
